# Segment Occlusion vs. Reconstruction—A Single Center Experience With Endovascular Strategies for Ruptured Vertebrobasilar Dissecting Aneurysms

**DOI:** 10.3389/fneur.2019.00207

**Published:** 2019-03-13

**Authors:** Stefan Schob, Anett Becher, Pervinder Bhogal, Cindy Richter, Anna Hartmann, Katharina Köhlert, Felix Arlt, Svitlana Ziganshyna, Karl-Titus Hoffmann, Ulf Nestler, Jürgen Meixensberger, Ulf Quäschling

**Affiliations:** ^1^Abteilung für Neuroradiologie, Universitätsklinikum Leipzig, Leipzig, Germany; ^2^Department of Interventional Neuroradiology, The Royal London Hospital, London, United Kingdom; ^3^Klinik für Neuroradiologie, Klinikum Stuttgart, Stuttgart, Germany; ^4^Klinik und Poliklinik für Neurochirurgie, Universitätsklinikum Leipzig, Leipzig, Germany; ^5^Klinik und Poliklinik für Anästhesiologie und Intensivtherapie, Universitätsklinikum Leipzig, Leipzig, Germany

**Keywords:** dissecting aneurysm, vertebral artery, subarachnoid hemorrhage, flow diverter, stent assisted coiling, segment occlusion

## Abstract

**Objective:** Ruptured dissecting aneurysms of the intracranial vertebral arteries exhibit an extraordinarily high risk for morbidity and mortality and are prone to re-rupture. Therefore, early treatment is mandatory to induce stagnation of the critical dynamic mural process. Appropriate endovascular approaches are segment sacrifice and reconstruction, however, both carry specific risks and benefits. To date most studies discuss only one of these approaches and focus on one specific device or technique. Therefore, our study aimed to present our experiences with both techniques, providing a considered approach on when to perform endovascular reconstruction or sacrifice.

**Materials and Methods:** We retrospectively reviewed patients with subarachnoid hemorrhage in our database, suffering from dissecting aneurysms of the intradural vertebral arteries and treated endovascularly in the acute setting. A total of 16 cases were included. Clinical history, radiologic findings and outcomes were analyzed.

**Results:** In 7 patients a reconstructive approach was chosen with 4 of them receiving stent-assisted coiling as primary strategy. One of the 7 patients suffered early re-bleeding due to progression of the dissection and therefore treatment was augmented with implantation of 2 flow diverters. The remaining 2 patients were primarily treated with flow diverters in telescoping technique. In 9 patients a deconstructive approach was followed: 6 patients were treated with proximal coil-occlusion of the V4 segment, 3 patients received distal coiling of the V4 segment. Two patients died (GOS 1) in the subacute stage due to sequelae of recurrent episodes of raised intracranial pressure and parenchymal hemorrhage. Two patients kept severe disability (GOS 3), six patients had moderate disability (GOS 4) and seven patients showed full recovery (GOS 5). None of the patients suffered from a procedural or postprocedural ischemic stroke.

**Conclusions:** In patients with good collateral vascularization, proximal, or distal partial segment sacrifice via with endovascular coil occlusion seems to yield the best risk-benefit ratio for treatment of ruptured dissecting V4 aneurysms, especially since no continued anticoagulation is required and possibly essential surgery remains feasible in this scenario. If possible, PICA occlusion should be avoided—although even proximal PICA occlusion can become necessary, when weighing against the risk of an otherwise untreated ruptured V4 dissecting aneurysm. Contrarily, if the dominant V4 segment is affected, the hemodynamic asymmetry prohibits occlusion and necessitates reconstruction of the respective segment. For this, implants with high metal coverage treating the entire affected segment appear to be the most promising approach.

## Introduction

Arterial dissections in the domain of the vertebrobasilar territory are an overall rare but important cause for ischemic and hemorrhagic stroke in young patients (1). As described in earlier studies, the anatomical disposition of the terminal vertebral artery segments and their remarkable hemodynamic profile facilitate incremental loss of structural integrity of the local vessel wall and thus constitute a unique vulnerability of the V4 segments for mural injury (2, 3). Accidental intimal damage subsequently allows blood to enter the vessel wall with high velocity and pressure, dissecting the wall along its layers, resulting in the formation of a false lumen or intramural hematoma (4). Depending on their exact localization and extent vertebral artery dissections may very rarely occur without apparent symptoms, infrequently manifest as subarachnoid hemorrhages but mostly cause neck- and occipital pain, which is frequently followed by posterior circulation stroke (5). If a dissection is located extra-cranially and follows a predominantly sub-intimal course, a variable degree of steno-occlusive disease develops and causes cerebral ischemia in up to 60% of all patients (6, 7). However, the risk for recurrent stroke decreases considerably after 10 days and the overall prognosis with conservative medical treatment is comparatively good (8). If the intimal tear primarily affects the intracranial segment or continues intra-cranially through the dural entry point and then further extends sub-adventitially, a dissecting aneurysm evolves and may rupture primarily or secondarily, thus leading to subarachnoid hemorrhage (SAH) (9). Mortality in these patients can be dramatically high, with rates up to 83% (10). Also, early rebleeding has been reported to occur in 9 of 10 patients within 24 h after the initial hemorrhagic event (11). As a consequence, dissecting aneurysms of the intracranial posterior circulation must be treated interventionally as fast as possible to prevent further hemorrhage and the higher morbidity associated with repeat rupture.

Therapy options involve both—open neurosurgical and endovascular approaches. Surgically, the afflicted segment can be treated via bypass, trapping or clipping (12–14). However, endovascular approaches are favored since open surgery carries a high risk of lower cranial nerve palsy and brainstem injury (15, 16). Furthermore, surgery related morbidity is inherently higher compared to the minimally invasive transarterial technique (17).

Similar to the neurosurgical approaches, the endovascular treatments comprise deconstructive and reconstructive techniques (17). In the past, endovascular segment occlusion (parent vessel sacrifice, trapping) using detachable coils has been performed successfully with high rates of permanent occlusion, reliably preventing rebleeding (14, 17). Unfortunately, this approach carries a significant risk of ischemic stroke related to perforators of the occluded segment and to hemodynamic changes in the dependent PICA territory (14, 18). Therefore, endovascular reconstruction of dissected vertebral arteries has been performed more recently, using a stent-in-stent technique, stent-assisted coiling or employing flow diverting stents with the aim to prevent ischemic sequelae whilst restoring original hemodynamics (10, 19–22). As a limitation, all of the reconstructive techniques require a relevant period of dual antiplatelet therapy in the setting of SAH, which may jeopardize further neurosurgical interventions, such as external ventricular drainage or craniectomy.

Considering the currently available studies, evidence regarding the safety and efficacy of the two endovascular regimes is limited to comparatively small case studies focusing on either of them. More specifically, no clear clinical guideline is available indicating when to preferably occlude or rather reconstruct a vertebral artery carrying a dissecting V4 aneurysm. Therefore, this study aimed to present our experience with patients treated for dissecting distal vertebral artery aneurysms in an acute SAH setting, especially considering the actual necessity for vessel preservation in context of the individual hemodynamic situation at hand.

## Materials and Methods

The study with retrospective design was approved by the Institutional Ethics Committee (local IRB nr. AZ 208-15-0010062015). Informed consent of each patient regarding the scientific use of radiological and clinical data was obtained in writing either from the patient himself or his / her legal representative.

Our institutional radiology information system database was searched for acute SAH cases associated with vertebrobasilar dissection in the period of January 2010 to May 2018. SAH severity was graded according to the initial CCT using the Fisher scale (23). Each patient's clinical status ad admission was assessed and documented using the Hunt and Hess grading system (24). The latest outcome was assessed in the neurovascular outpatients clinic or at the time point of discharge from our hospital.

### Diagnostic Angiography and Interventional Strategy

Diagnostic digital subtraction angiography (DSA) was performed using either a biplane Siemens system (Axiom Artis, Erlangen, Germany) or a biplane Philips system (Allura, Best, The Netherlands). A dissecting vertebral artery aneurysm was diagnosed as underlying cause for SAH in case one or a combination of the following morphological features was visible on angiography: focal dilatation, string sign, sudden luminal tapering, string and pearls, fusiform aneurysmal dilatation or vessel irregularity (25–27). All patients were treated under general anesthesia. To prevent catheter associated thromboembolic events, an initial bolus dose of 5,000 i.v. heparin was given intravenously.

Depending on the individual location of the dissection in context of the specific collateral circulation either a deconstructive (proximal or distal V4 occlusion, *n* = 9) or a reconstructive endovascular therapy (*n* = 7) was performed. Procedural details and patients characteristics are summarized in Table 1 (deconstructive group) and Table 2 (reconstructive group) A detailed description of the respective interventional techniques is provided in Supplementary File A. Supplementary File B demonstrates the corksrew-coiling technique.

**Table 1:** Summarizes demographic and clinical aspects of patients treated via a deconstructive approach.

**Case**	**Sex**	**Age (years)**	**Location; hemodynamic situation**	**Fisher grade**	**Hunt & hess**	**Lesion dimensions in mm**	**Pseudo-aneurysm max. diameter in mm**	**Endovascular approach**	**EVD**	**Craniectomy**	**DSA follow up**	**Clinical follow up**	**Pre-OP GCS**	**GOS time of review**
1	Female	68	Left hypoplastic V4; no significant posterior communicating artery, dominant right vertebral artery	4	4	11.5 × 4	4	Proximal segment sacrifice	Left frontal	No	No	8 months	3	4
2	Female	55	Left hypoplastic V4; no significant posterior communicating artery, dominant right vertebral artery	4	4	15 × 3	3	Proximal segment sacrifice	Left frontal	No	3 months after	3 months, then annually	6	5
3	Female	76	Co-dominant left V4 with equally important right V4; fetal type posterior cerebral artery	4	4	18 × 2	2	Proximal segment sacrifice	Left frontal	No	No	3 months	12	5
4	Female	52	Right hypoplastic V4; dominant left V4, small posterior communicating artery	4	3	18 × 3	3	Proximal segment sacrifice	No	No	No	6 months	13	5
5	Female	61	Left hypoplastic V4; dominant right vertebral artery, fetal type posterior cerebral artery	4	2	10 × 2	2	Proximal segment sacrifice	No	No	No	4 months	12	5
6	Male	55	Right co-dominant V4 with equally important left V4, no significant posterior communicating artery	3	3	20 × 7	5	Proximal segment sacrifice	No	No	no	6 months	8	4
7	Male	54	Right co-dominant V4;equally strong left V4, no significant posterior communicating artery	4	4	30 × 5	18	Distal segment sacrifice	No	No	No	1 month	6	1
8	Female	66	Left co-dominant V4 with equally strong right V4, small posterior communicating artery	3	4	20 × 4.2	4.2	Distal segment sacrifice	Right frontal	Yes	No	2 months	10	4
9	Female	81	Right co-dominant V4 with equally important left V4; no significant posterior communicating artery	4	4	15 × 3	3	Distal segment sacrifice	Bifrontal	No	No	2 months	7	5

**Table 2:** Summarizes demographic and clinical aspects of patients treated via a reconstructive approach.

**Case**	**Sex**	**Age (years)**	**Location, hemodynamic situation**	**Fisher grade**	**Hunt & hess**	**Lesion dimensions in mm**	**Pseudo-aneurysm max. diameter in mm**	**Endovascular approach**	**EVD**	**Craniectomy**	**DSA follow up**	**Clinical follow up**	**Pre-OP GCS**	**GOS time of review (months)**
1	Male	53	Left dominant V4; hypoplastic right V4, strong posterior communicating artery	4	2	15 × 7	10	Stent-assisted Coiling (LEO)+ Flow Diverter (2 × p64)	Right frontal	No	14 days	2 months	14	4
2	Male	70	Right dominant V4, hypo-plastic left V4, strong posterior communicating artery	4	4	30 × 10	10	Stent-assisted Coiling (Enterprise)	Right frontal	No	No	4 months	7	4
3	Female	59	Left dominant V4, hypoplastic right V4, no significant posterior communicating artery	3	1	2.1 × 0.3	8	Stent-assisted coiling (Enterprise)	None	No	1, 3 and 4 years	3 months, then annually, latest 5 years after SAH	13	5
4	Female	51	Right codominant V4 with equally strong left V4, no significant posterior communicating artery	4	4	20 × 5	8	Stent-assisted coiling (Enterprise)	Right frontal	No	No	5 months	8	5
5	Female	57	Left dominant V4, hypoplastic right V4, small posterior communicating artery	4	2	15 × 4.3	2.5	Flow Diverter (2 × p64)	None	No	4 months after SAH	5 months	12	5
6	Female	53	Left dominant V4, right hypoplastic V4, strong posterior communicating artery	4	4	29 × 7	7	Flow Diverter (3 × PED2 Flex)	Right frontal	No	No	1 month	7	4
7	Female	68	Left dominant V4, hypoplastic right V4, small posterior communicating artery	4	3	28 × 6	6	Flow Diverter (1 × PED 2 Flex)	Right frontal	Yes	No	6 months	6	1

The clinical status after treatment was assessed in the neurosurgical department using the Glasgow outcome scale (GOS) 1-5 after a median time of 4 months (minimum 1 month, maximum 8 months), 1 representing death and 5 representing good recovery.

## Results

After thorough review of clinical and radiological data, 16 patients were identified with acute SAH from vertebrobasilar dissecting aneurysms. Nine patients were treated with either proximal or distal V4 occlusion, while seven patients were treated with a reconstructive approach using either stent assisted coiling or the implantation of flow diverting stents. Table 1 provides information on the deconstructively treated collective and Table 2 summarizes information of the reconstructively treated patients. Additionally, 11 cases of conclusive intracranial vertebral artery dissection not manifesting with SAH but provoking ischemic posterior circulation stroke were identified, but not included in the study. Spontaneous vertebrobasilar dissections rarely occur after the 6th decade. However, the oldest individual in our patients collective was 81 years old. This advanced age is *per se* associated with especially high mortality and morbidity after SAH. Also, considering the aforementioned demographic normality, some of the acutely ruptured pseudoaneurysms may well have been the result of earlier vertebral artery dissections, suggesting the possibility not only of early, but also years later SAH after initial vertebral artery intimal injury. One of the cases was associated with a low velocity motorcycle accident; the remaining cases had no history of preceding trauma. All patients presented to our emergency department, were treated within 24 h of admission to our neurosurgery department and were admitted to our neurointensive care unit for post treatment surveillance.

### Procedural Results

In 9 patients decision was made for a deconstructive approach. Of those, 6 patients were treated with proximal segment occlusion and 3 received distal segment closure.

**Figure 1:**
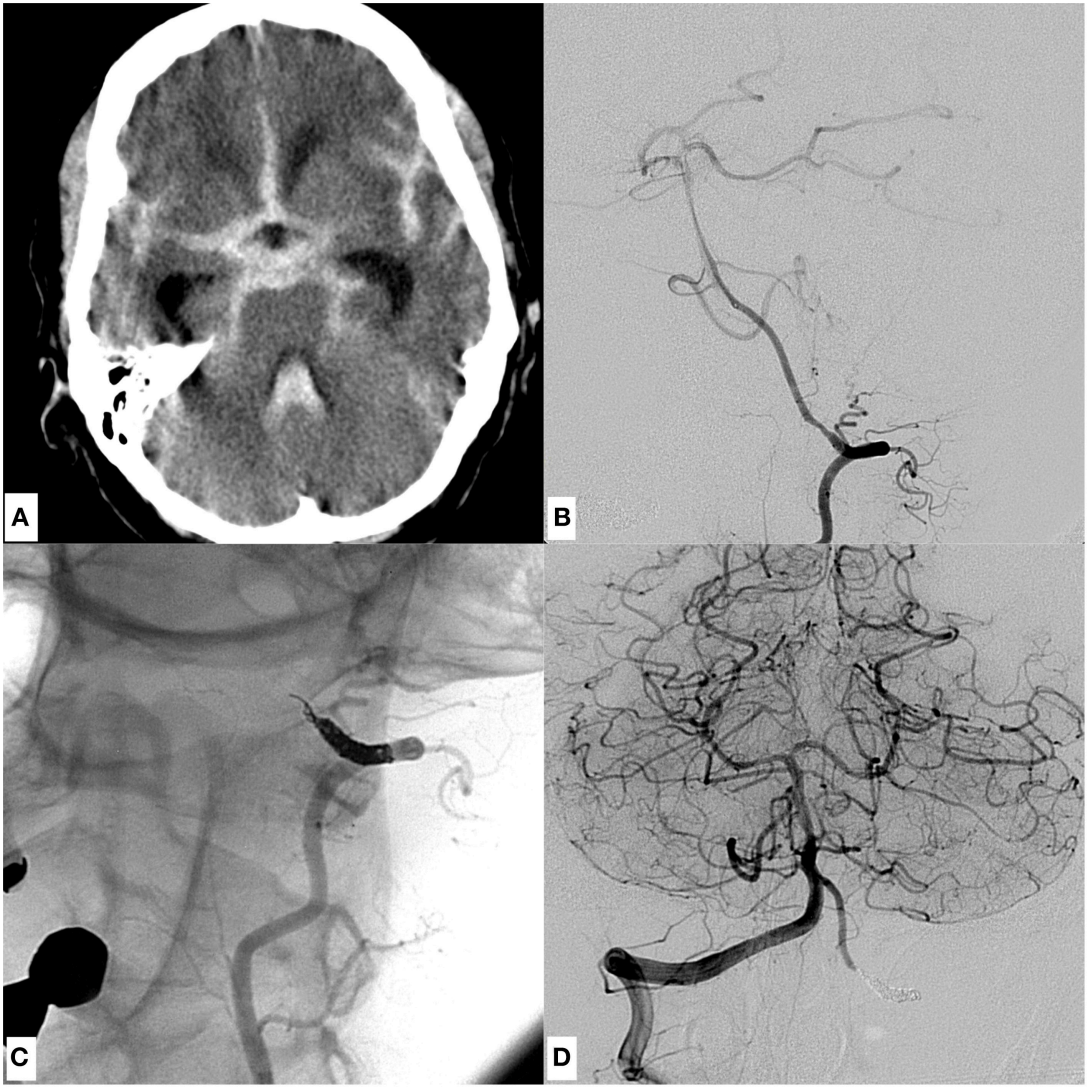
An example of a dissecting V4 aneurysm causative for SAH Fisher grade 4, Hunt and Hess grade 4, treated with proximal endovascular segment occlusion. **(A)** Shows a representative slice of the initial CCT with significant SAH. **(B)** Demonstrates the mural injury of the hypoplastic left-sided intradural vertebral artery, visible as tapered narrowing of the proximal V4 segment in an oblique projection of a left vertebral artery injection. **(C)** Image after proximal segment occlusion in magnified working projection: two coil loops of the first coil were anchored intraluminally behind the distal end of the dissected V4 segment in corkscrew technique, preventing retrograde reperfusion of the dissection site. Note the elliptically shaped, condensed proximal coil bundle which occludes the proximal starting point of the dissection, solidly impeding its anterograde reperfusion. **(D)** Depicts the right vertebral artery angiogram, showing distinct retrograde perfusion of the remaining left V4 segment, thus securing sufficient supply of small brain stem perforators. Symmetric contrast filling of both cerebellar hemispheres directly after coiling, indicating sufficient blood flow into the entire posterior circulation through the unaffected right vertebral artery. The patient did not suffer rebleeding and showed excellent recovery 3 months after the event (GOS 5).

**Figure 2:**
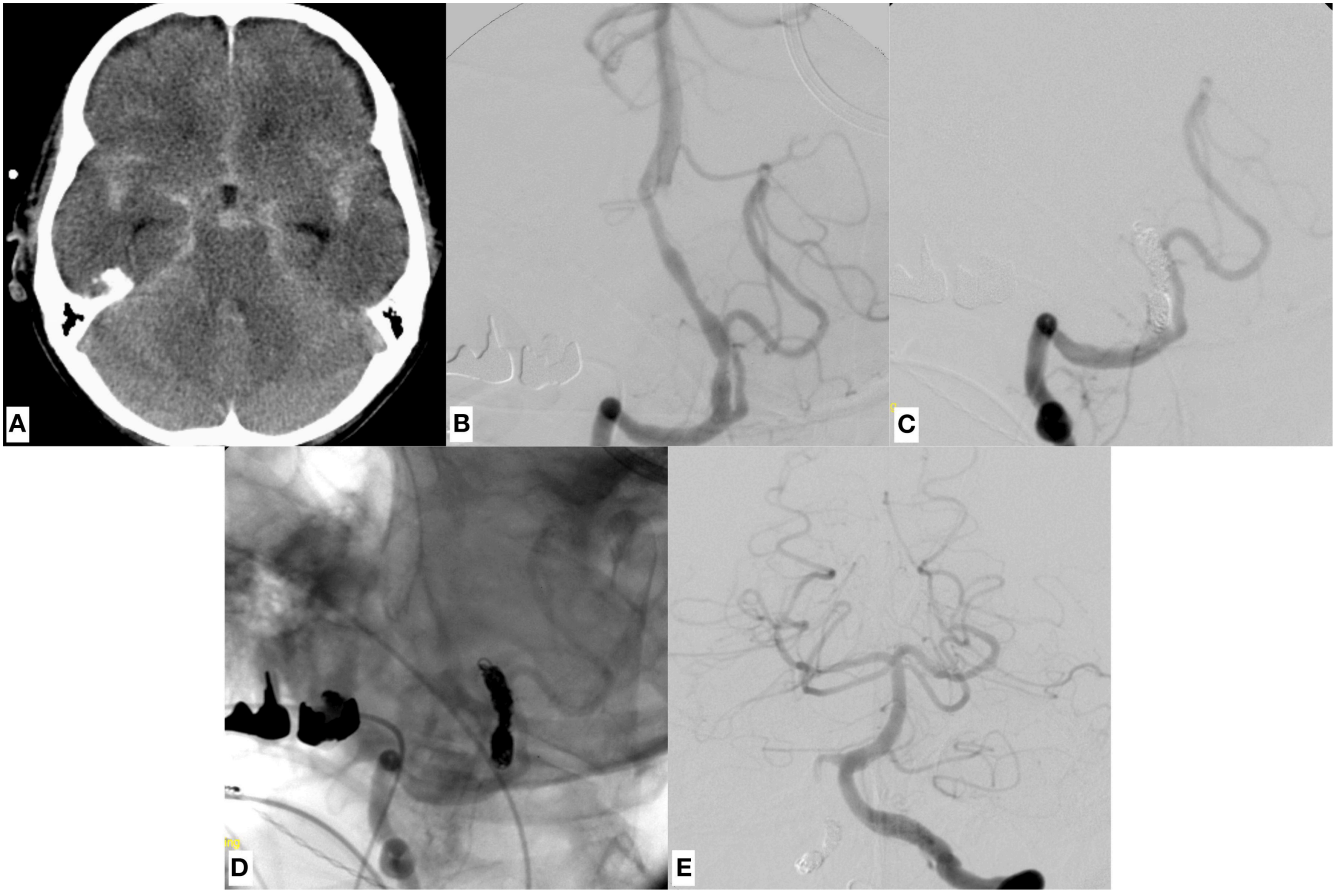
A representative case of a dissecting V4 aneurysm (SAH Fisher grade 4, Hunt and Hess grade 4) of the right sided, small-sized vertebral artery, treated with distal endovascular segment occlusion. **(A)** Shows the initial CCT revealing SAH. **(B)** Depicts the right vertebral artery pathology, visible as pearl and string sign in DSA. Note the significant PICA branching proximal to the affected V4 portion. **(C,D)** Provide substracted and unsubstracted images of right vertebral artery injections after distal segment occlusion, leaving the PICA orifice open. Strong opacification of the now functionally PICA-terminating V4 segment. The first coil, which was anchored in corkscrew technique within the true lumen of the distal V4, is securing the dissecting aneurysm whilst impeding anterograde or retrograde reperfusion. **(E)** The contralateral vertebral artery angiogram, showing symmetrical opacification of the entire posterior territory after occlusion of the right-sided dissected V4 segment. The patient did not suffer rebleeding and showed excellent recovery 2 months after the event (GOS 5).

**Figure 3:**
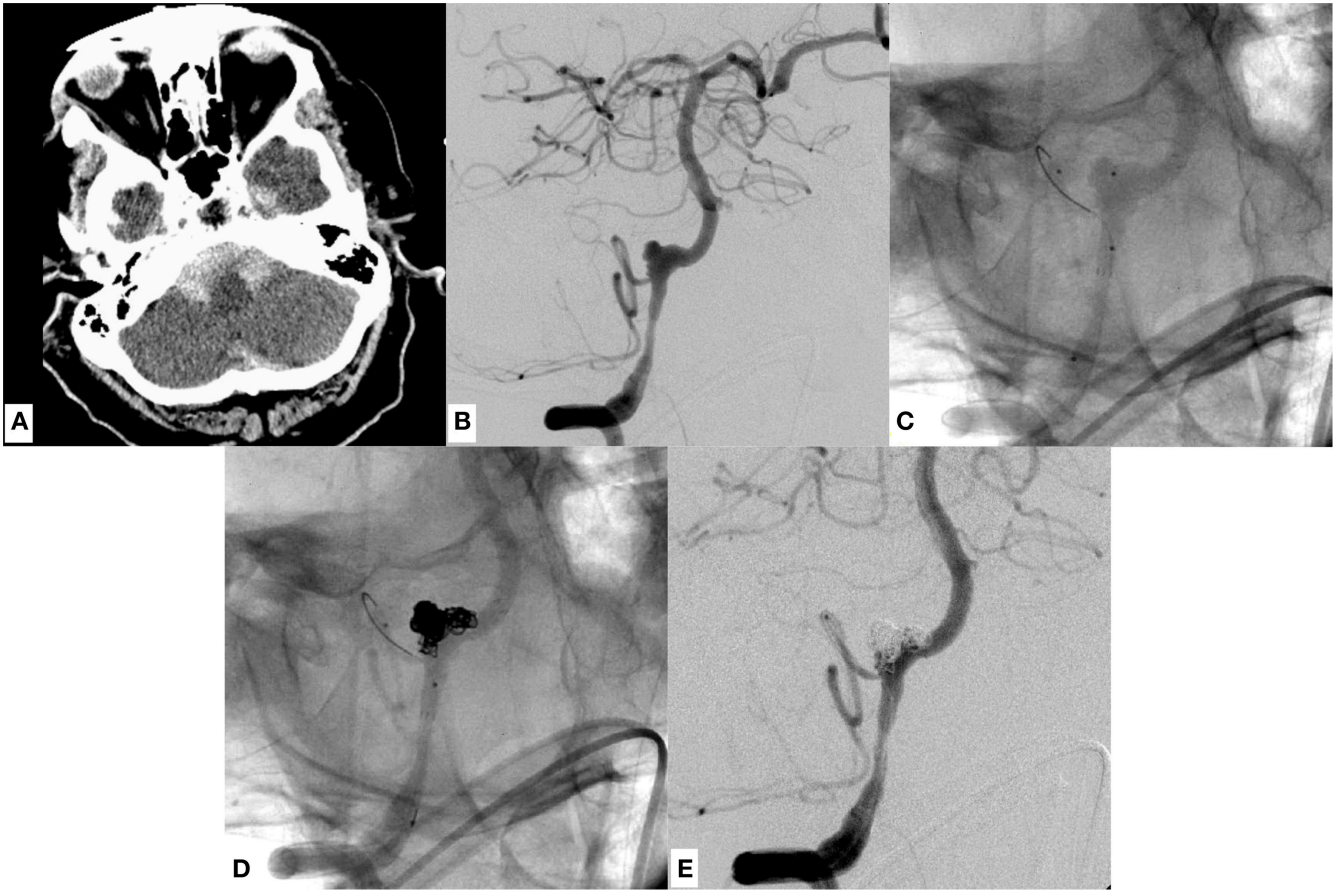
An example of SAH due to a ruptured dissecting aneurysm of the right sided, hemodynamically dominant vertebral artery involving the PICA orifice. It was finally managed through stent assisted coiling using a laser cut device (Enterprise stent, Codman Neuro, USA). **(A)** Demonstrates basal SAH. **(B)** Shows a DSA image of a right vertebral artery injection revealing the causative large dissecting aneurysm, which was treated with Enterprise stent—assisted coiling **(D)** in jailing technique. **(C)** Radiopaque markers of the enterprise stent proximal and distal to the aneurysm. **(D,E)** Show the successful reconstruction of the affected V4 segment with continuous opacification of the PICA. Note the placement of the micro-guide-wire in the proximal PICA. **(C–E)** The patient regained self-reliance in the daily routine (GOS 4) 4 months after the event.

In 7 patients a reconstructive approach was followed. Figure 1 provides an exemplary case of proximal V4 occlusion. Figure 2 demonstrates a case of distal V4 occlusion. To reconstruct the affected segment, the following devices were used: 3 × Enterprise stent, 1 × Leo+ stent, 4 × Pipeline Embolisation Device Flex 2, and 3 × p64. An example of stent-assisted coiling for the reconstruction of a dominant V4 segment dissecting aneurysm is shown in Figure 3. In this case a laser cut stent (Enterprise stent) and the Micrus coil system were used. For this, the coiling catheter was initially placed into the dissecting aneurysm and jailed by carefully deploying the Enterprise stent across the opening of the intimal flap. The stent not only acts to seal the flap but also helps to prevent coil protrusion into the true lumen and provides a rather subtile degree of flow diversion. This occurs by the induction of numerous eddy currents in the outer layers of the bloodstream, evoked by contact of the rapidly streaming blood with the coarse mesh of the device, hereby altering the parabolic profile of blood flow.

**Figure 4:**
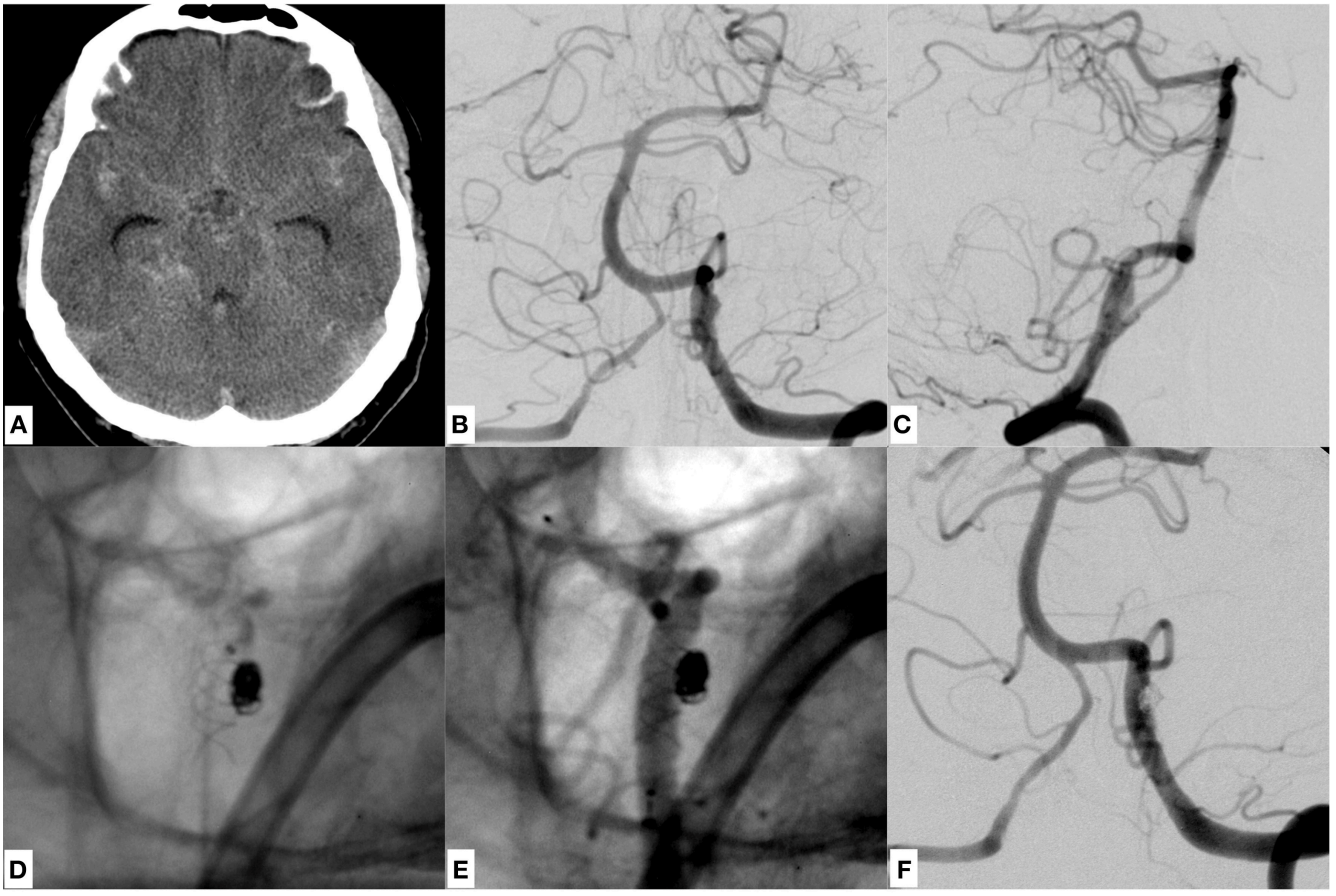
A case of SAH due to a ruptured dissecting aneurysm of the dominant left sided vertebral artery of a 53 years old male patient, which was initially treated with stent assisted coiling using a braided stent (LEO, Balt, France). **(A)** Provides a representative CCT section showing SAH Fisher grade 4. **(B,C)** Give the anteroposterior and lateral DSA images revealing the underlying, morphologically subtle dissecting aneurysm close to the PICA orifice. The inferior row of images **(D–F)** shows the LEO stent and the adjacent coil bundle after treatment *in situ* without **(D)** and with injection of contrast agent **(E)**, demonstrating unremarkable vertebrobasilar opacification **(F)** (see Figure 5).

**Figure 5:**
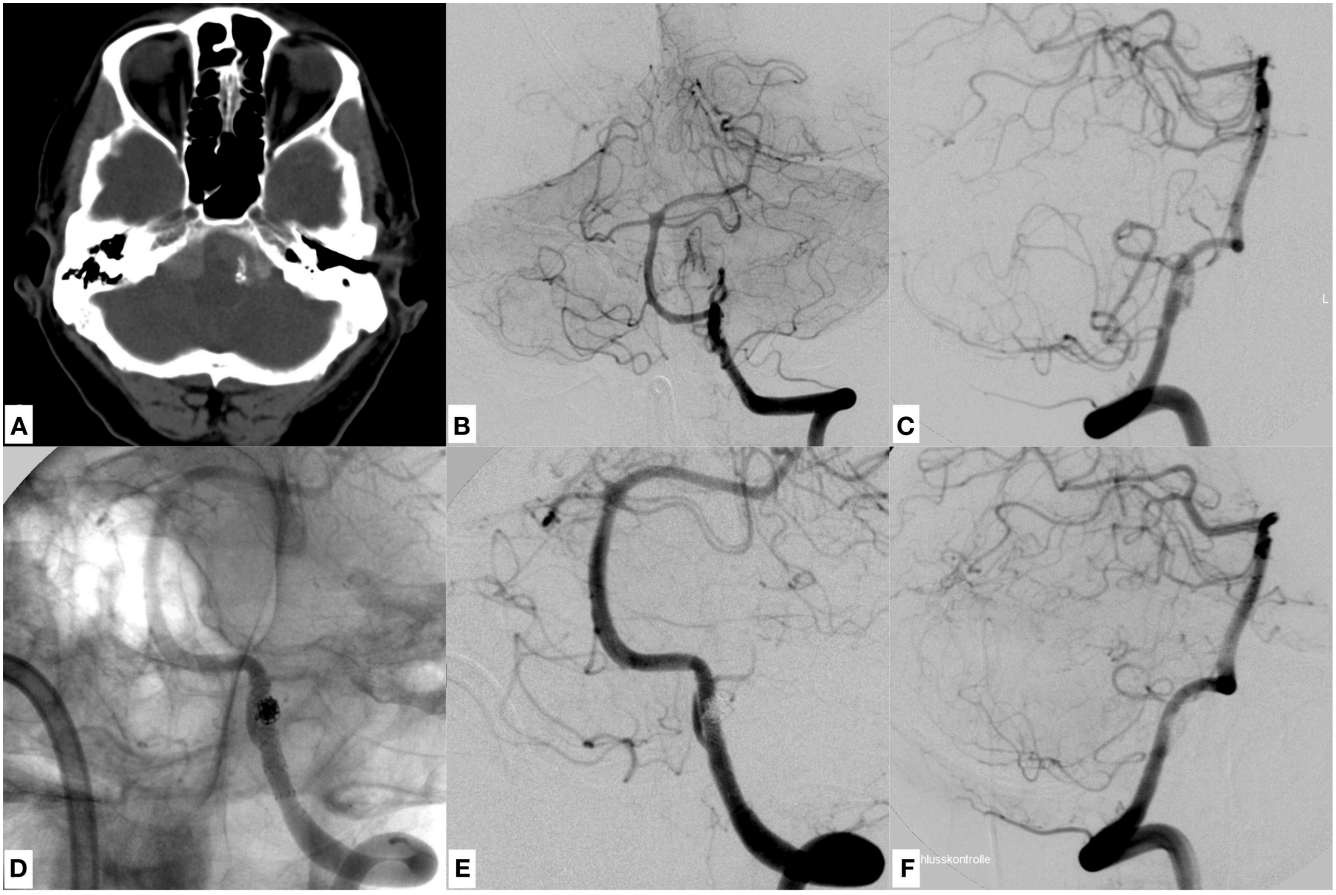
The complicated further course of the patient initially treated with stent assisted coiling (Figure 4). 7 days after the first procedure the patient complained of sudden, predominantly left sided, most severe head and neck pain. Therefore, a CCT was performed **(A)** and showed a significant subarachnoid rebleeding predominantly in the basal cisterns. Note the clearly depictable radiopaque LEO stent in the left V4 segment and the surrounding hyperdense subarachnoid blood. Images **(B,C)** show the morphologically subtle progress of the previously treated dissecting aneurysm of the left V4 segment. To secure the further distended dissecting aneurysm—whose proximal and distal endings were not precisely definable—two overlapping flow diverters (p64, Phenox, Germany) with highly increased surface coverage in comparison to the priorly implanted LEO stent were used to safely overlay the whole affected segment **(D)** in telescoping technique. Note the eight circular radiopaque markers at the proximal ending of both flow diverters in **(D)**, which are now spanning the proximal and distal end of the previously implanted LEO stent. Images **(E,F)** show the successfully reconstructed vertebral artery with delayed but complete filling of the ipsilateral PICA. The patient did not suffer further rebleeding and regained self-reliance in the daily routine (GOS 4) 2 months after the event.

**Figure 6:**
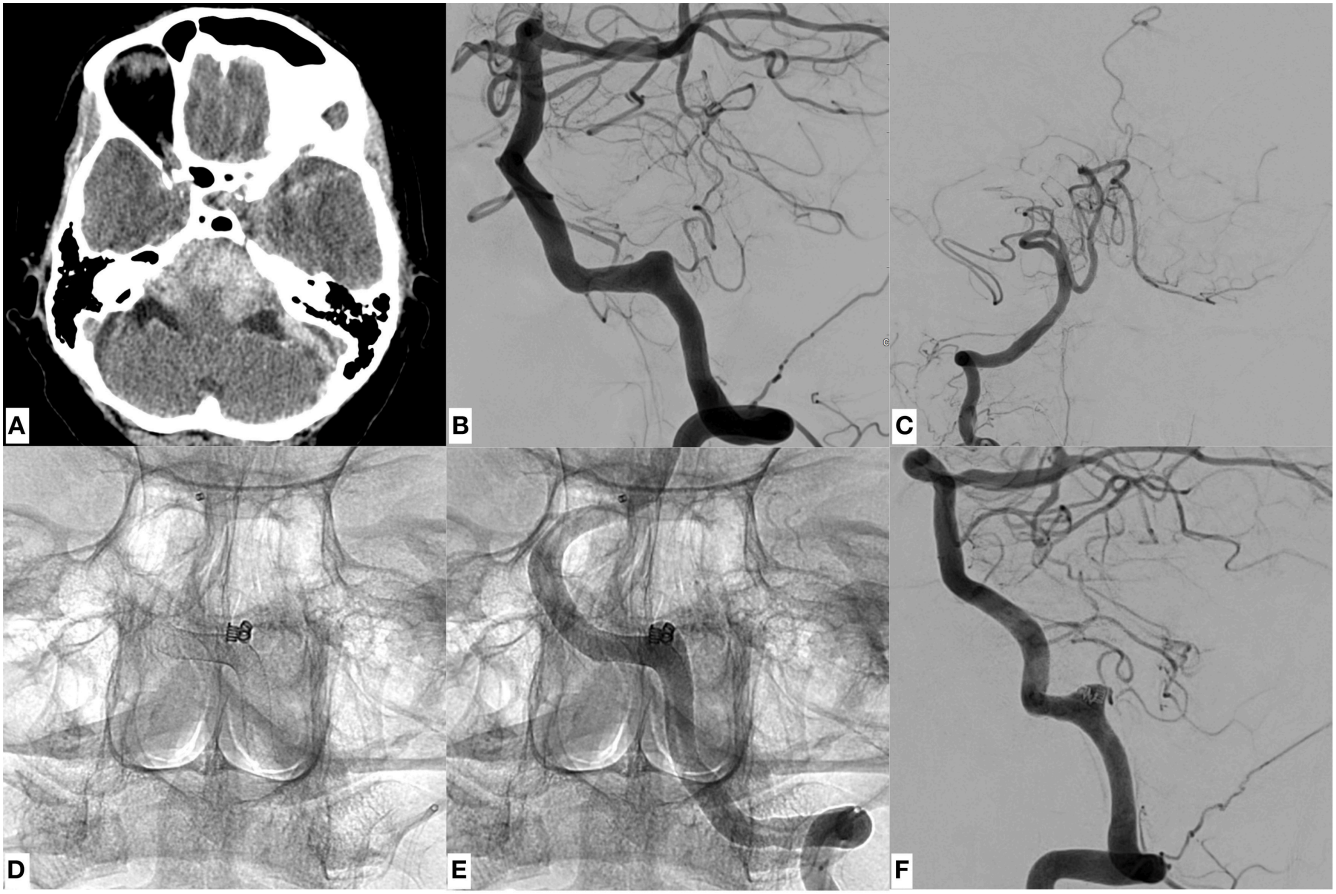
A patient being treated with 3 flow diverters plus coiling in jailing technique (Fisher 4, Hunt and Hess 4). CCT **(A)** had shown a significant subarachnoid hemorrhage with accentuation in the posterior fossa. Image **(B)** shows the underlying dissecting aneurysm of the left sided, dominant vertebral artery located in the terminal V4 segment. **(C)** PICA-terminating contralateral vertebral artery, without sufficient collateral circulation to the posterior circulation. To promote coagulation within the pseudoaneurysm, 2 coils were placed carefully within the aneurysm sac after implantation of 3 overlapping PED 2 Flex flow diverters **(D,E)**. The dissecting aneurysm was highly fragile as indicated by the subtle growth of the false lumen during the intervention (most obvious when comparing the morphology of the pseudoaneurysm in **(B,E)**. **(F)** Provides the final injection after successful reconstruction of the segment. The patient did not suffer further rebleeding and is recovering from the hemorrhagic event (GOS 4).

Figure 4 shows the reconstructive approach for a hemodynamically indispensable V4 segment affected by a large dissecting aneurysm using a braided nitinol device, the LEO+, which exhibits a higher surface coverage than the Enterprise stent, in combination with Micrus coils. Comparable to the case in Figure 3, the coiling catheter was placed into the pseudoaneurysm sac and then jailed by the LEO stent before the subsequent coil occlusion. Figure 5 demonstrates the continued endovascular treatment after progress and rebleeding of the dissecting aneurysm displayed in Figure 4, seven days following initial treatment. Two flow diverters, characterized by the highest surface coverage of all used devices (p64) were employed in an overlapping manner (telescoping technique) within the LEO-stent carrying segment. With this telescoping flow diverters-in-stent approach, the highly dynamic dissecting aneurysm was secured and no further episode of hemorrhage occurred. Figure 6 demonstrates a case of endovascular reconstruction of a dissected segment employing overlapping flow diverters in the first place. As the placement of intracranial stents—most importantly of densely woven flow diverters—necessitates dual platelet inhibition to prevent ischemic complications (in-stent thrombosis, thromboembolic stroke), the following pharmacological regimen was applied: on the day of the procedure 500 mg ASA were given I.V. during the intervention, accompanied by a bolus of 180 μg/kg eptifibatide I.V. before stent placement, immediately followed by post-operative loading with 300 mg clopidogrel. For the post-treatment period 100 mg ASA and 75 mg Clopidogrel were given orally each day and continued for 6 months if an Enterprise or Leo stent were implanted and 12 months if a flow diverter was implanted.

### Further Course and Outcome

One of seven patients in the reconstructive group suffered re-bleeding after initial reconstruction with a LEO stent-assisted coiling due to progressive dissection and necessitated additional endovascular treatment (Figures 4, 5). This was performed using two p64 flow diverters in telescoping technique and resulted in complete reconstruction of the vessel without further harmful sequelae. Three Patients were treated with Enterprise stent-assisted coiling, one was treated with a singular flow diverter (p64) one was treated with 2 flow diverters in telescoping technique primarily (PED 2 flex) and one was treated with 3 flow diverters (PED 2 flex). None of these six patients did experience rebleeding. One fatality (GOS1) occurred in the reconstructive group. The patient had been treated with a p64 in the acute phase, but continued to show enlargement of ventricles on CT-scan after removal of the external ventricular drainage. A continuous lumbar cerebrospinal drainage was initiated, but failed to restore CSF circulation sufficiently. As ventricular enlargement progressed rapidly, a repeat external ventricular drainage placement was performed under double anticoagulatory treatment. This was followed by massive recurrent subcutaneous and intracerebral hemorrhages, finally leading to decompressive craniotomy. The patient succumbed to the hemorrhage related herniation.

There was no rebleeding in the deconstructive occlusive group or procedure related complication. Furthermore, there was no procedural or postprocedural ischemic stroke, due to thromboembolism or perforator occlusion in those 9 patients. One of the patients in the deconstructive group died after repeated recurrent episodes of highly elevated, uncontrollable intracranial pressure closely shortly after the initial hemorrhage (GOS 1). Two patients had kept severe disability (GOS 3), six patients had moderate disability (GOS 4) and seven patients had showed full recovery (GOS 5)

Direct follow up DSA was available for the 7 cases with vessel reconstruction, our only recently adopted follow-up strategy now comprises examinations 3 and 9 months after intervention, in the remaining deconstructive cases the follow-up angiographic controls are at 6 and 24 months after occlusion.

## Discussion

Endovascular techniques have evolved as the treatment of choice for dissecting aneurysms of the distal vertebral arteries associated with SAH. However, no consistent strategy regarding when to reconstruct or when to occlude an affected V4 segment has been presented yet. Most published studies in this context favor one specific technique or a singular device and do not balance shortcomings with advantages in an equilibrated fashion. Hence, our study aimed to present our experience with both—deconstructive and reconstructive—endovascular approaches in the acute SAH setting, proposing a considerate treatment algorithm.

As exemplified by Figures 1, 2, endovascular coil occlusion of the affected part of a small-sized or hypoplastic V4 segment for treatment of its dissecting aneurysm may be considered a worthwhile procedure, especially if occlusion of the PICA orifice is omittable or the size of the respective PICA territory is insignificant. Also, the presence of a hemodynamically significant posterior communicating artery, which in case of vertebrobasilar occlusion secures supply of the midbrain, must be included in the appreciation of the therapeutic algorithm.

In our patients, occlusion of the dissected part anterior to the PICA orifice was safely possible without any harmful procedure related sequelae and with good GOS outcome scores in all of the 6 patients (Table 1). In this way, flow reversal within the remaining V4 portion via the contralateral dominant vertebral artery was induced and the angiographically occult brain stem perforators were sustained or collateralized, as no brain stem infarction occurred.

On the other hand, if the dissecting V4 aneurysm is only affecting the terminal V4 segment of a relatively small or hypoplastic vertebral artery distal to the PICA orifice, respective segment occlusion is possible without harmful procedure-related sequelae. In this constellation, sufficient anterograde perfusion of the PICA territory is maintained through the proximally unaffected vertebral artery, and perfusion of the remainder of the posterior intracranial circulation is delivered by the dominant contralateral vertebral artery. Also, the presence of a significant posterior communicating artery, ensuring supply of the midbrain via the anterior circulation in case of vertebrobasilar occlusion, must be considered in this context. In the present study, a good GOS was reached in 1 from 3 patients (Table 1).

This rationale is in line with the basic idea of Madaelil et al.'s study, suggesting that endovascular parent vessel sacrifice, especially if good collateral circulation is at hand, represents a safe and viable therapy option for dissecting V4 aneurysms (14). Even in cases where the PICA cannot be spared, the benefit of protective coil occlusion entailing PICA ischemia seems to outweigh the high risk for mortality and severe morbidity as a consequence of rehemorrhage following treatment omission (28, 29). This consideration is corroborated by the fact that significant PICA infarctions are encountered only rarely after proximal PICA occlusion, as pial collaterals from adjacent branches like the SUCA and AICA often equalize supply of the PICA territory (14).

However, V4 segments and PICAs regularly give rise to relevant brain stem supplying perforating arteries (30) and medullary infarction caused by perforator occlusion as a consequence of endovascular segment occlusion has been reported to be associated with poor outcomes (31). None of our patients developed brain stem ischemia. As demonstrated by Aihara et al. (32) the risk for medullary infarction associated with distal segment sacrifice is generally very low (ca. 6%), although the symptoms, if manifested, are severe (26). In contrast to this, the probability for brain stem infarction can be as high as 50% in proximal segment occlusion, with symptoms being commonly mild. In our cohort, three patients received distal segment occlusion, considered to be at low risk for brain stem infarction.

Six patients received proximal segment occlusion. In four of those the affected segment belonged to a unilaterally hypoplastic vertebral artery, which in <30% of all cases gives rise to relevant perforating arteries (33). Thus, the absence of brain stem infarction in our deconstructively treated cohort relies on a favorable anatomical constitution in this regard. To our minds, the application of a precise corkscrew coiling technique substantially contributes to the prevention of perforator related brain stem infarction. The technique ensures the shortest possible segment occlusion by precisely obturating the dissection only, and hereby avoids unnecessary occlusion of precedingly unaffected perforators.

In patients where the dissected V4 segment is rated hemodynamically indispensable, for example if the contralateraI V4 is terminating as PICA or it is significantly narrower than the affected vessel (18), reconstruction of the primary brain stem supplying V4 segment is inevitable (especially if no strong posterior communicating artery ensuring midbrain perfusion in case of vertebrobasilar occlusion is present). In scenarios of this sort, restoration can be achieved using a stent-in-stent technique, stent assisted coiling or the application of flow diverters (10, 19–22), notwithstanding that the use of the latter is most favorable, as increased endovascular surface coverage has been demonstrated to be associated with lower rates of re-bleeding and recurrence of dissecting aneurysms (20–22).

From a technical point of view, in a number of cases the precise entry point and—most importantly—the exact dimension of the dissection remain inconclusive in DSA, since the vessel wall is not adequately evaluable using catheter based angiography (34). Even MRI does not necessarily reveal the detailed intramural pathology during the acute phase, because initial signal intensities of mural hematomas are rather similar to the adjacent tissue and only just become increasingly distinct within the following weeks (35). As a consequence, the neurointerventionalist may not perform the endovascular reconstruction in a sufficient length, which in this case results in inappropriate covering of the entire dissection, allowing for further incremental mural destruction and leading to rehemorrhage. Hence, the combined use of a number of devices may become necessary to completely cover the dissected segment and secure the aneurysm (Figure 4).

However, endovascular reconstruction inevitably exerts significant mechanical forces on the *per se* fragile arterial wall segment and thus inherently carries a higher risk for rehemorrhage blood extravasation during the intervention, namely rebleeding (36, 37). Furthermore, a certain period of dual platelet inhibition is essential to prevent ischaemic complications secondary to implant-associated coagulation. This in turn increases the severity of hemorrhagic complications during ventriculostomy, vp-shunt insertion, hemicraniectomy and tract bleeding as well as the hemorrhagic transformation of subacte ischemic areas exemplarily occurring from vasospasm.

Limitations of our study are the retrospective design and the analysis of only a small number of patients, not providing enough statistical power to conduct a comparative calculation regarding patient outcomes and procedural complications. Secondly, long-term follow-up DSAs are not yet performed in all cases and are restricted to patients that are returning for evaluation to our outpatient clinic.

Also, vertebrobasilar dissections rather occur beyond the 6th decade. Our collective includes a number of significantly older patients. This may only be a peculiarity of our study. However, it may indicate the delayed formation of critical pseudoaneurysms being responsible for SAH even years after the initial vascular injury. Hence, the indication for treatment of intracranial vertebral artery dissections should be made also accounting for the aforementioned possibility.

### Outlook

Coil occlusion of an intracranial vertebral artery as one approach is associated with significant, most importantly ischemic complications and also may—if not performed sufficiently—allow for delayed antegrade or retrograde perfusion of the dissection. In our experience, a precise corkscrew coiling technique reduces the risk for ischemic complications and reperfusion of the dissected segment:

At first, few loops of the initial coil are carefully anchored in the previously, micocatheter-injection confirmed true lumen, which directly borders the distal end of the mural defect. The anchoring area distal to the wall injury within the adjoining healthy segment must be kept as short as possible to minimize the probability for occlusion of (yet unaffected) perforators. Then, the coil is delicately condensed within the mural defect in proximal direction to efficiently seal the distal portion of the dissection and thereupon prevent its retrograde reperfusion. For this purpose, an utterly compliant, long coil claiming only a minimal diameter must be employed to avoid unnecessary transmural force. Finally, the coil packaging is completed in proximal direction. This way it is sealing the entry site of the dissection and prevents its anterograde reperfusion.

Finally, based on the relative rarity of the condition, experience with vertebral artery dissection related SAH remains small even in large neurovascular centers. Therefore, we ask for the participation of all neurointerventionalists in a conjoint, international registry of vertebrobasilar dissection associated SAH.

## Conclusions

Endovascular occlusion of a hypoplastic or small-sized V4 segment for treatment of its dissecting aneurysm is a safe option, does not require potentially harmful, long-lasting anticoagulation and should be preferred over of a complex endovascular reconstruction, associated with the potential risk of recurrence and rebleeding.

Although it is certainly desirable to preserve the PICA orifice, considering the hazards associated with non-treatment, even proximal occlusion of the dependent PICA can be considered to be an acceptable alternative. Nevertheless, it is essential to keep the occluded section as short as possible to avoid infarction of angiographically occult brain stem perforators.

**Figure 7:**
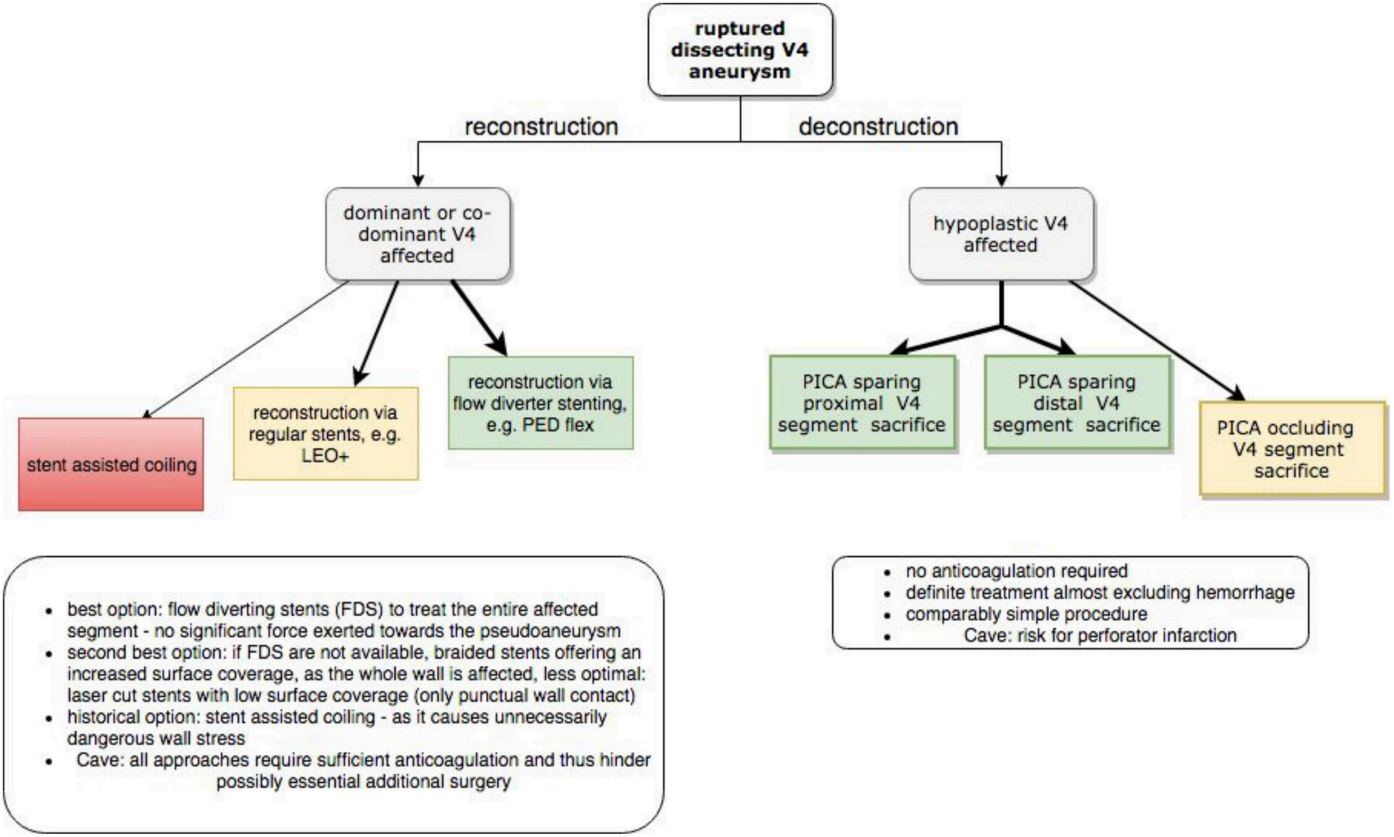
Interventional algorithm for endovascular treatment of dissecting V4 aneurysms especially considering the hemodynamic situation at hand. Green panels represent the most favorable option. Arrow thickness indicates higher preferability of the respective approach.

If the affected V4 segment is hemodynamically dominant, reconstruction is inevitable and should be performed with devices offering the highest possible surface coverage, as this significantly reduces the risk for rebleeding. Unfavorably, this scenario requires a significant period of dual anti-platelet medication, which impedes potentially life-saving neurosurgical interventions as external ventricular drainage or craniotomy and may exacerbate hemorrhagic complications. Thus, the individual hemodynamic situation, preexisting comorbidities and the potential need for further neurosurgery determine which endovascular approach is most appropriate for treatment of a ruptured dissecting V4 aneurysm. Figure 7 provides a strategic roadmap which graphically summarizes the pre-interventional considerations to identify the most suitable approach.

## Author Contributions

SS and UQ performed endovascular treatments and drafted the paper. AB, PB, CR, AH, SZ, KK, FA, UN, K-TH, and JM participated in case management and performed review of the individual files. FA, UN, KK, and JM performed neurosurgical interventions. SZ was responsible for intensive care management and anesthesia.

### Conflict of Interest Statement

The authors declare that the research was conducted in the absence of any commercial or financial relationships that could be construed as a potential conflict of interest.
