# Supplementary material for: Segment Occlusion vs. Reconstruction—A Single Center Experience With Endovascular Strategies for Ruptured Vertebrobasilar Dissecting Aneurysms
Source: Front Neurol. 2019 Mar 13;10:207. doi: 10.3389/fneur.2019.00207 (PMC6424888; doi:10.3389/fneur.2019.00207)
Supplement: Supplementary file 1 [file Data_Sheet_1.docx]

**Supplement A – description of endovascular techniques**

*Deconstructive approaches*

In cases where the dissected V4 segment did not involve the PICA branching and appeared to be hypoplastic on angiography, it was rated as hemodynamically expendable (n=9). Then, a deconstructive approach was followed and the segment was occluded endovascularly by coiling. For this, either the Micrus Endovascular Coil System (Codman Neuro, Johnson & Johnson, USA) or the Target Detachable Coil System (Stryker Neurovascular, USA) was applied. Since the length of the affected segment to be occluded is directly linked to the risk of perforator ischemia, the occlusion was kept as short as possible (28). Table 1 summarizes patients in the deconstructively treated group.

*Proximal V4 segment sacrifice*

More specifically, if the entire affected segment was localized proximal to the PICA branching, the segment was occluded from the distal end of the dissection to the presumed proximal opening of the false lumen, pursuing a corkscrew coiling technique while precisely sparing the PICA orifice. For this, the first loop of the coil was anchored within the distally adjoining true lumen, which was initially confirmed by microcatheter injection. Then, the coil was deployed under careful retraction in proximal direction, forming a dense coil bundle which safely corked the highly fragile dissecting aneurysm, finally anchoring the last coil loop in the proximally adjacent true lumen. By application of this technique, only the actual dissecting aneurysm became occluded and its antegrade or retrograde reperfusion were prevented most effectively. As a consequence, retrograde flow through the contralateral vertebral artery into the remaining distal V4 portion was preserved. Figure 1 gives an example of proximal V4 occlusion, allowing for retrograde perfusion of the unaffected remaining distal V4 segment supplying the PICA. The corkscrew technique is furthermore illustrated in supplementary figure 8.

*Distal V4 segment sacrifice*

In cases where the entire affected segment was located distal to the PICA orifice, distal coil occlusion using the corkscrew technique was performed. For this, the peripheral true lumen was probed and the first coil loop was anchored there, carefully omitting the V4 confluens. The coil bundle was subsequently developed and condensed in proximal direction as described above. The final coil loop was eventually placed into the previously confirmed true lumen, proximally bordering the dissecting aneurysm and sparing the PICA orifice. Figure 2 provides an example of distal V4 occlusion, allowing sufficient antegrade perfusion of the ipsilateral PICA.
In both deconstructive groups, no platelet function inhibitory drugs were given.

*Reconstructive approach - V4 reconstruction*

If the affected V4 segment belonged to a hemodynamically indispensable vertebral artery (n=7), e.g. carrying a strong PICA or representing the dominant vertebral artery (thus giving rise to the majority of local perforating branches) combined with a contralateral, hypoplastic vertebral artery, a reconstructive approach was pursued. Prior to the availability of flow diverters in our institution, reconstruction was performed using either lasercut stents (Enterprise stent: Codman Neuro, Johnson & Johnson USA) or braided stents (LEO stent: Balt, France) in combination with the previously mentioned coil systems. As soon as flow diverters became available in our center, they were exclusively employed for the reconstruction of dissecting V4 aneurysms. The Pipeline Embolisation Device Flex 2 (PED2: Medtronic, USA) or the p64 (Phenox, Germany) were applied in this context. To sufficiently treat dissecting V4 aneurysms of hemodynamically dominant vertebral arteries, the true lumen of the whole morphologically conspicuous segment was covered with the respective devices, in order to completely seal the intimal flap, provide internal stabilization to the injured segment and prevent intraluminal coil protrusion or transmural migration over the course of mural consolidation. To obtain disruption of intra-aneurysmal flow and acceleration of coagulation within the pseudoaneurysm sac, the latter was loosely filled with coils, carefully avoiding any punctual mechanical force exerted on the fragile vessel wall. Table 2 summarizes patients being treated with V4 reconstruction.
