# Supplementary material for: Segment Occlusion vs. Reconstruction—A Single Center Experience With Endovascular Strategies for Ruptured Vertebrobasilar Dissecting Aneurysms
Source: Front Neurol. 2019 Mar 13;10:207. doi: 10.3389/fneur.2019.00207 (PMC6424888; doi:10.3389/fneur.2019.00207)

**Supplement B**

Supplement B illustrates the corkscrew coiling technique. At first, few loops of the initial coil are carefully anchored in the previously, micocatheter-injection confirmed true lumen, which directly borders the distal end of the mural defect. The anchoring area distal to the wall injury within the adjoining healthy segment must be kept as short as possible to minimize the probability for occlusion of (yet unaffected) perforators. Then, the coil is delicately condensed within the mural defect in proximal direction to efficiently seal the distal portion of the dissection and thereupon prevent its retrograde reperfusion. For this purpose, an utterly compliant, long coil claiming only a minimal diameter must be employed to avoid unnecessary transmural force. Finally, the coil packaging is completed in proximal direction. This way it is sealing the entry site of the dissection and prevents its anterograde reperfusion. (A) shows the right vertebral artery injection, demonstrating the dissecting V4 aneurysm. (B) and (C) show an early stage of coil occlusion. Note the distal anchoring of the first coil within the true lumen and the continuation of the coil into the mural defect, securing the distal end of the dissection. (D) and (E) demonstrate the final stage of corkscrew coiling with sufficient proximal and distal occlusion of the dissecting aneurysm. (F) demonstrates the injection of the contralateral VA with sufficient opacification of the unaffected small PICA.


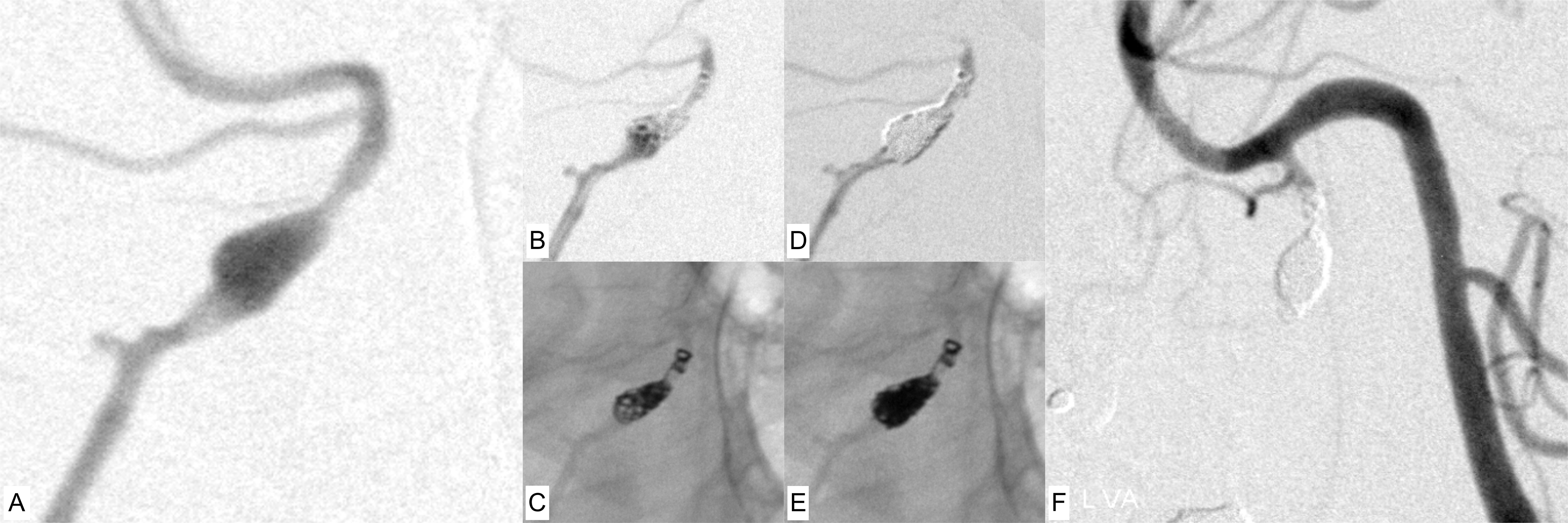

Supplement: Supplementary file 2 [file Data_Sheet_2.docx]
